# Advanced Dialogues: From Genomes to Microbiomes—A Cross‐Disciplinary Journey

**DOI:** 10.1002/ggn2.202500039

**Published:** 2025-06-28

**Authors:** Jingyuan Fu

**Affiliations:** ^1^ Department of Genetics University of Groningen University Medical Center Groningen Groningen 9712 the Netherlands; ^2^ Department of Pediatrics, University of Groningen University Medical Center Groningen Groningen 9712 the Netherlands

## 1 Introduction

My research group, Integrative Omics in Systems Medicine, is affiliated at the University Medical Center Groningen, the Netherlands. Our research aims to understand host‐microbe interactions in complex traits and diseases, for which we integrate genetics, microbiome, and large‐scale omics data to identify risk factors and their interactions underlying inter‐individual variation in disease susceptibility.

## 2 Challenges and Opportunities in Genetics and Genomics

The most pressing challenges lie in the undefined causality and molecular mechanisms underlying observed associations. In the post‐GWAS and big data era, many genetic loci, microbial species, and other risk factors have been linked to various phenotypes. However, the translation of these findings into personalized medicine remains limited. I see promising opportunities in two areas. First, recent advances in artificial intelligence (AI) are facilitating genome annotation, risk prediction, and drug target discovery. Second, microfluidic organ‐on‐a‐chip technologies, combined with the differentiation and culturing of human induced pluripotent stem cells (hiPSCs), enable the construction of individualized organ‐on‐a‐chip systems for studying disease mechanisms and testing drugs, all while accounting for a person's unique genetic background.

## 3 A Cross‐Disciplinary Journey

My scientific journey has three important turning points. I received my bachelor's degree in biochemistry and switched to the field of bioinformatics for my master's. This was the first turning point that enabled me to establish my knowledge in both molecular biology and bioinformatics. The second turning point was when I successfully completed my Ph.D. project in systems genetics using a plant model organism in 2007. At the time, the first draft sequence of the human genome was just published, and genome‐wide association studies (GWAS) began to emerge for genome‐wide genetic screening. I saw an opportunity to extend my expertise from plant genomics to human genetics. This chance permitted me to cross the disciplinary border toward the field of medicine. The third turning point took place in 2013 when I further extended my research area from human genetics to the human gut microbiome. The first microbiome study was published in *Circulation Research* in 2015^[^ ^1^ ^],^ which was highlighted in the *TIME* book “TIME 100 New Health Discoveries: How the latest breakthroughs affect your health and wellness”.^[^ ^2^ ^]^ Then in 2016, our first metagenome‐based study was published in *Science*, which was also highlighted on the cover.^[^ ^3^ ^]^ These two studies marked the beginning of my scientific journey in the microbiome field. Recently, our studies revealed inter‐individual differences in the gut microbiome and the underlying environmental and genetic factors^[^ ^4^, ^5^ ^]^, reported its temporal dynamics and stability,^[^ ^6^, ^7^ ^]^ its strain‐level genetic variants, as well as its interaction with genetics and diet in metabolic regulation.^[^ ^8^ ^]^ Therefore, looking back my scientific journey, my diverse research background demonstrates the importance of stepping out of one's comfort zone by engaging in cross‐disciplinary work.

## 4 The Power of Mentorship

Many individuals have shaped my journey. I am especially grateful to my postdoctoral supervisor, Prof. Cisca Wijmenga—a visionary scientist whose leadership and creativity have had a profound influence on my development. She initiated pioneering research lines in genetics, microbiome, and organ‐on‐a‐chip technologies at the University Medical Center Groningen. These fields, once perceived as emerging or high‐risk, have now become integral pillars of biomedical research and are central to major initiatives in precision medicine and systems biology.

Under her mentorship, I learned not only to think big and across disciplines but also to build bridges between fundamental science and translational applications. Her ability to identify promising directions ahead of time taught me the value of scientific courage, strategic vision, and collaborative leadership.

## 5 Navigating Work‐Life Balance as a Woman in Science

I often ask myself the same question, and there is probably no perfect answer. The pressure in academia is considerable, and the situation in China is even more severe than in the Netherlands.

I recently attended a TEDx talk by Kika Buhrmann, CEO of Nespresso Netherlands. She said that work is part of life, and that it's not about time management—after all, we all have the same amount of time. Instead, it's about energy management: how to manage your activities so that they give you energy rather than drain it. This reflects my approach as well. For example, it is important for me to take breaks of at least 1–2 h a day and longer ones after a period of intense work to recharge. I also keep a priority list—daily, weekly, and monthly— to help track progress and stay focused. And most importantly, I enjoy traveling, which helps me find balance and inspiration.

## 6 Advice to Early‐Career Researchers and Students

Science today is increasingly cross‐disciplinary. My advice to early‐career researchers and students is to stay curious, be bold, and embrace the unknown. Don't be afraid to step outside your comfort zone and think outside the box—this is often where the most exciting discoveries occur.

## 7 The Future is Collaborative

I strongly believe in TEAM science. Collaboration is extremely important, and working together across disciplines not only accelerates scientific progress but also enriches your own perspective. I am delighted to be able to work with a group of researchers at the Groningen Microbiome Hub who share a similar mission but with complementary expertise. For example, combining genetics with organ‐on‐a‐chip technologies enables functional validation of disease mechanisms in a human‐relevant context. Likewise, AI‐driven models are transforming how we predict genetic risk, interpret non‐coding variants, and discover drug targets. Such cross‐pollination of ideas is accelerating the transition from data‐rich to insight‐rich science.

## 8 Recognition and Collective Achievement

This recognition by the Royal Netherlands Academy of Arts and Sciences and the Royal Holland Society of Sciences and Humanities are deeply meaningful to me, and much of the credit goes to my team, colleagues, and collaborators. I am also grateful for the support from the University Medical Center Groningen, the University of Groningen, and the Lifelines Biobank—this achievement would not have been possible without them.

## 9 Shaping the Future of Publishing

I feel that genetics‐based journals underwent rapid growth in the past 20 years, along with the development of the genetics and genomics research fields. However, the field of genetics is currently encountering a bottleneck in the transition from association studies to translational research. I think “*Advanced Genetics*” should focus on the term “Advanced”: building a platform for cutting‐edge and innovative breakthroughs. To better serve researchers and practitioners, I believe the journal should continue to evolve in several ways. First, they can promote more open and transparent science by supporting preprints, data sharing, and reproducible research practices. Second, they can actively encourage cross‐disciplinary submissions that reflect the complexity of modern research. Third, journals can play a stronger role in mentoring the next generation of scientists by inviting them to join the Youth editorial board and offering them opportunities to bring their vision on future research to the field.

This article was edited by Yuming Hu, Wiley.

## Conflict of Interest

The author declares no conflict of interest.
